# A comprehensive protocol for evaluating health, safety, and environmental risks of hospital solid waste through FMEA technique

**DOI:** 10.1016/j.mex.2024.102760

**Published:** 2024-05-14

**Authors:** Towhid Dadashi, Saeed Hosseinpoor, Amir Mohammadi

**Affiliations:** a Department of Environmental Health Engineering, School of Public Health, Urmia University of Medical Sciences, Urmia, Iran; b Social Determinants of Health Research Center, Clinical Research Institute, Urmia University of Medical Sciences, Urmia, Iran

**Keywords:** Hazardous waste, HSE risk assessment, FMEA technique, Hospital Solid Waste HSE Risk Assessment protocol using FMEA technique

## Abstract

This protocol outlines a comprehensive approach to evaluating hospital solid waste levels and assessing associated health, safety, and environmental (HSE) risks using the Failure Mode and Effects Analysis (FMEA) methodology. The study focuses on Imam Khomeini Hospital (RA) and employs both quantitative and qualitative methods. Over a 3-month period, waste production and potential risks are assessed, with specific attention to household, infectious, medicinal, and sharps waste. Through FMEA, potential failure modes and associated risks in waste management sectors are identified, enabling targeted interventions for risk mitigation. The protocol emphasizes the importance of aligning waste management practices with international standards and highlights the need for comprehensive training, awareness campaigns, and effective waste management methods to ensure the safety and environmental responsibility of hospital waste management practices

## Graphical abstract

**Figure:**
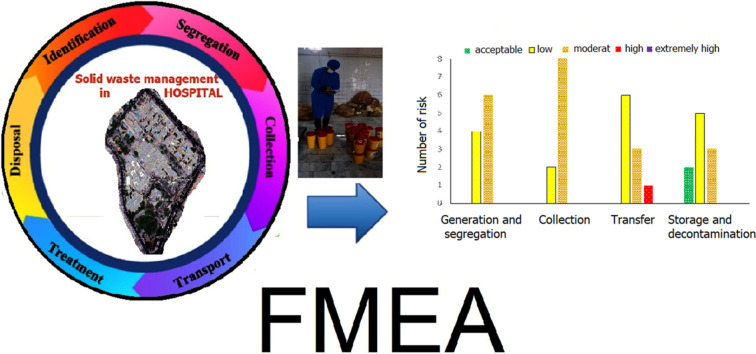

Specifications table

**Table:** 

Subject area:	Environmental Health Engineering
More specific subject area:	Hazardous waste management
Name of your protocol:	Hospital Solid Waste HSE Risk Assessment protocol using FMEA technique
Reagents/tools:	Observation, Interview templates, Excel program, FMEA checklists
Experimental design:	The experimental design involves a comprehensive approach to assess hospital waste management HSE risks. It begins with a preliminary investigation, identifying stakeholders and departments. Observation and interviews identify risks. FMEA is applied for systematic risk assessment, determining severity, occurrence, and detection levels. Risk priority numbers (RPN) categorize risks. Disposal methods are implemented per waste category. Data analysis involves extracting parameters and calculating RPN. Quality assurance and control ensure accuracy. Documentation includes findings and recommendations. Continuous improvement integrates insights for risk mitigation and process enhancement
Trial registration:	Not applicable
Ethics:	The current research was funded by Urmia University of Medical Sciences, West Azerbaijan, Iran (grant no: 3199), and adhered to ethical standards outlined in ethics code IR.UMSU.REC.1402.008. All participating workers and additional staff provided informed consent during the study. The authors express appreciation to Urmia University of Medical Sciences and all contributors to the research.
Value of the Protocol:	• Comprehensive assessment of hospital solid waste and associated health, safety, and environmental risks. • Utilization of Failure Mode and Effects Analysis (FMEA) methodology for systematic risk identification and mitigation. • Emphasis on aligning waste management practices with international standards and promoting safety and environmental responsibility.

## Background

The protocol provided here stems from a pressing global challenge surrounding the management of hospital solid waste, amidst rapid urbanization and industrialization. It is well-documented that ineffective waste management practices pose substantial risks to both public health and the environment. Against this backdrop, the Failure Mode and Effects Analysis (FMEA) methodology emerges as a robust approach for identifying and mitigating potential failure modes and associated risks within complex operational frameworks. This protocol is motivated by a synthesis of recent research that has underscored shortcomings in hospital waste management practices, ranging from inadequate staff training to challenges in hazardous waste handling. Moreover, the protocol addresses the critical need for comprehensive waste management frameworks to ensure environmental sustainability and public health safety within healthcare settings [1].

By employing the FMEA technique, this study aims to proactively identify critical failure modes and potential risks within the medical waste management process, thereby illuminating the intricate network of hazards and failure points inherent within the system. Through systematic risk assessment and prioritization, the protocol facilitates the formulation of robust risk management strategies and proactive mitigation measures. The integration of FMEA enables the identification and prioritization of potential failure modes and risks, offering insights crucial for the development of enhanced operational protocols, advanced safety measures, and comprehensive contingency plans [2].

The novelty of this research protocol lies in its response to the urgent global challenge of hospital solid waste management amidst rapid urbanization and industrialization. It recognizes the documented risks ineffective waste management poses to both public health and the environment. Through the application of the Failure Mode and Effects Analysis (FMEA) methodology, the protocol aims to identify and mitigate potential failure modes and associated risks within complex operational frameworks. Unlike previous studies, this protocol synthesizes recent research to address shortcomings in hospital waste management practices, such as inadequate staff training and challenges in hazardous waste handling. Furthermore, it emphasizes the critical necessity for comprehensive waste management frameworks to ensure environmental sustainability and public health safety within healthcare settings. By conducting systematic risk assessment and prioritization, this protocol provides essential insights for developing improved operational protocols, advanced safety measures, and comprehensive contingency plans. In doing so, it fills a significant gap in the existing literature and makes a substantial contribution to the field.

## Description of protocol

Hospital waste management is a critical aspect of healthcare operations, with potential implications for both public health and environmental safety. However, there is a lack of standardized protocols for assessing and mitigating health, safety, and environmental (HSE) risks associated with hospital waste management practices. The Failure Mode and Effects Analysis (FMEA) methodology has emerged as a robust tool for systematically identifying and prioritizing potential failure modes and associated risks within complex operational frameworks. By developing a comprehensive protocol using FMEA, this study aims to fill this gap by providing researchers and practitioners with a replicable framework for assessing and managing HSE risks in hospital waste management. This protocol will contribute to enhancing the safety and sustainability of hospital waste management practices, ultimately improving public health outcomes and environmental protection [3,4].

### Step-by-step description of the protocol

**Fig. 1:**
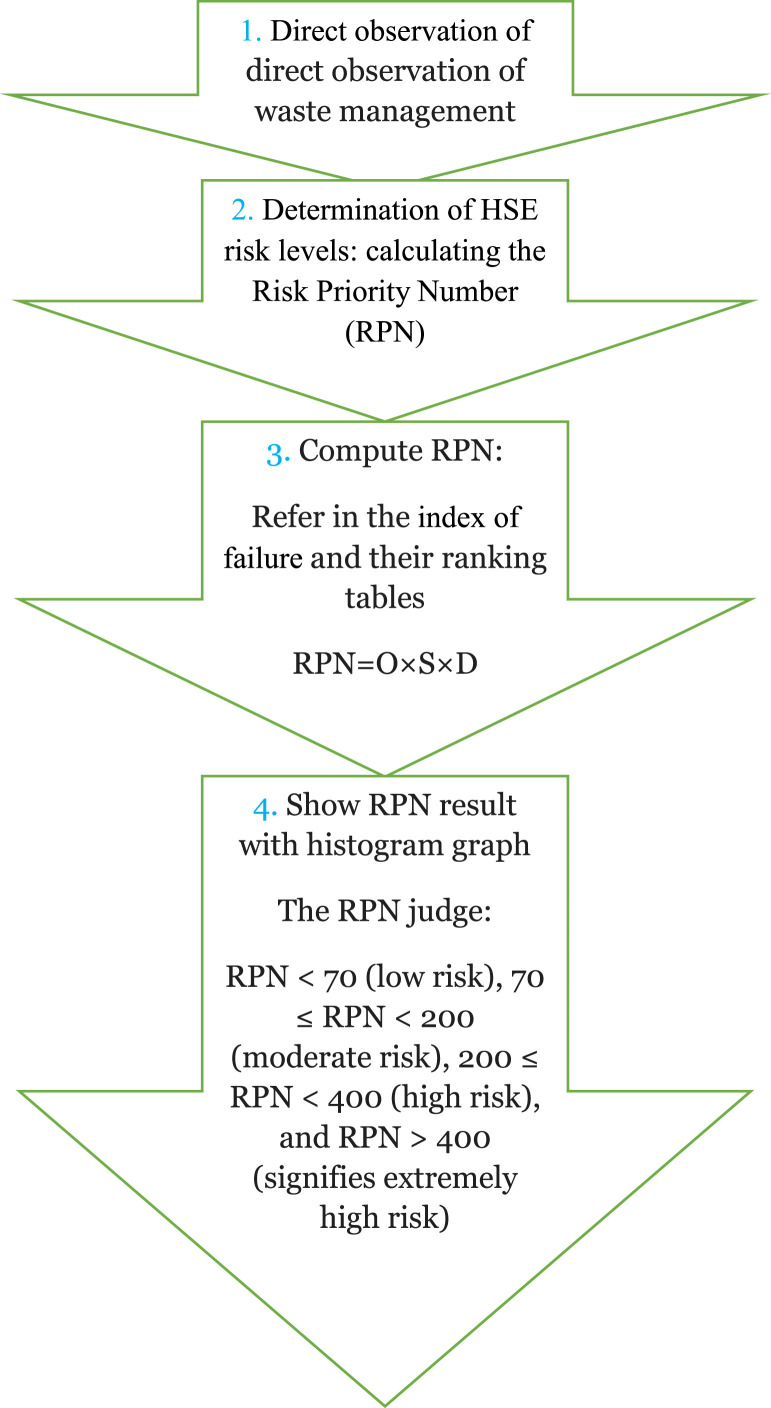
Flow diagram of FMEA steps for determination of HSE risks.

In the initial stage, as depicted in Fig. 1, a flow diagram illustrates the process undertaken to identify significant Health, Safety, and Environment (HSE) risks in hospital solid waste management using FMEA methodology.

### Preliminary investigation

Conduct a thorough assessment of the hospital waste management system to understand its organizational structure and processes. Identify key stakeholders and personnel involved in waste management.

### Recognition of hospital waste management departments

Map out the different departments responsible for managing hospital waste, including collection, segregation, transportation, and disposal.

### Identification of HSE risks

To identify health, safety, and environmental (HSE) risks related to hospital waste management, a multifaceted approach was employed. This involved direct observation of waste management processes, conducting structured interviews with key personnel involved, and employing comprehensive checklists to systematically assess potential hazards and risks.

### Risk assessment using FMEA

For systematic evaluation of HSE risks in hospital waste management, the Failure Mode and Effects Analysis (FMEA) methodology was adopted. FMEA worksheets were utilized to identify and analyze failure modes, their potential impacts, and the likelihood of their occurrence within the waste management framework.

Determine severity, occurrence, and detection levels for each identified failure mode.

### Determination of HSE risk levels

The determination of HSE risk levels involved calculating the Risk Priority Number (RPN) for each identified failure mode. This computation integrated multiple factors, including the probability of occurrence, severity of potential effects, and detectability of failures, thus providing a comprehensive assessment of risk levels.

Categorize RPN levels into four classes: low risk, moderate risk, high risk, and extremely high risk.

### Final disposal methods

Implement specific disposal methods for rendered wastes based on waste categories, ensuring compliance with recommended procedures and regulations.

The FMEA technique, a powerful risk assessment tool, employed a risk priority number based on occurrence, severity, and detection parameters [3,5]. For data analysis, which was calculated by Eq. (1):

(1) RPN=O×S×D

RPN: risk priority number, O: probability of occurrence, S: severity of effect, D: detectability

The occurrence, severity, and detection parameters were determined based on data extracted from Table 1, Table 2, Table 3. In fact, each parameter “occurrence, severity, and detection” was allocated an associated quality factor determined by the activity nature, as outlined in Table 1, Table 2, Table 3. The numeric result derived from Eq. (1) indicates the RPN level. The RPN number is categorized into four classes as follows: RPN < 70 is considered low risk, 70 ≤ RPN < 200 is categorized as moderate risk, 200 ≤ RPN < 400 indicates high risk, and RPN > 400 signifies extremely high risk.

**Table 1:** Ranking the likelihood (O - Occurrence) index of failure [6].

Order (O)	Criterion: the proportion of potential failure / total number of working days.	Likelihood of failure
10	O ≤ 1: 2	Extremely high
9	O ≤ 1: 10	Extremely high
8	O ≤1: 20	High
7	O ≤ 1: 100	High
6	O ≤ 1:200	Moderate
5	O ≤ 1: 1000	Moderate
4	O ≤ 1: 2000	Somewhat minimal
3	O ≤ 1: 10000	Low Extremely
2	O ≤ 1: 20000	low
1	O ≤ 1: 50000	Infrequent

**Table 2:** Assigning a rank to the S (severity) index of failure [6].

Order (S)	Criterion severity	Impact
9 ≤ S ≤ 10	Causing fatalities or complete system breakdown	Lethal
8 ≤ S ≤7	Inflicts severe harm to individuals or has a substantial impact on the system.	More detrimental
6 ≤ S ≤5	Results in lesser harm or a reduced impact on the system. Less	Less detrimental
4 ≤ S ≤ 3	Signifies a significant impact on individuals or the system with complete recovery.	Moderate
S = 2	Causes minimal disruption to the system or individuals	Low
S = 1	no effect on people or the system	No effects

**Table 3:** Rating for the capability to detect failures (Detection) [6].

Order (D)	Identifiable percentage	ID
10	Completely unknown	0 ≤ D ≤ 5
9	Very detailed	6 ≤ D ≤ 15
8	Partial	16 ≤ D ≤ 25
7	Very little	26 ≤ D ≤ 35
6	Low	36 ≤ D ≤ 45
5	Moderate	46 ≤ D ≤ 55
4	Moderately high	56 ≤ D ≤ 65
3	high	66 ≤ D ≤ 75
2	too high	76 ≤ D ≤ 85
1	Almost known	86 ≤ D ≤ 100

### Result of case study in one of Urmia hospital in northwest Iran

**Fig. 2:**
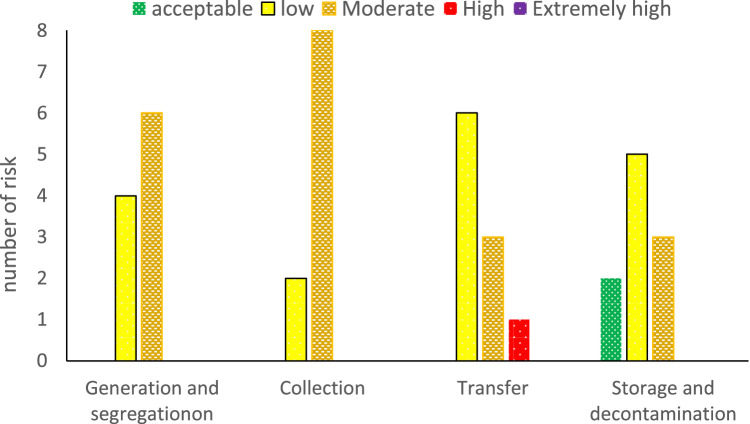
The outcomes of the health and safety risk assessment in different waste wards using the FMEA method [6].

In the HSE risk assessment conducted at Urmia Hospital utilizing the FMEA technique, each of the four stages of waste management (generation and segregation, collection, transfer, storage, and decontamination) revealed specific health and safety risks. The assessment identified a total of 10 risk items associated with various activities in each stage. The highest risk scores were observed in the transfer stage, categorized as high risk, while the lowest risk scores were found in the storage stage, classified as low or acceptable. No activity was classified as very high risk, but several activities were deemed high or moderate risk. The analysis pinpointed issues such as inadequate personnel training, low awareness levels, staff shortages, and lack of diligence as significant contributors to identified risks. Overall, the transfer stage emerged as the most critical phase with high-risk levels, while other stages exhibited acceptable, low, and moderate risks as Fig. 2. More detail given in our previous study [6].

## Conclusion

This protocol addresses the pressing challenge of hospital solid waste management by employing the Failure Mode and Effects Analysis (FMEA) methodology. In response to documented deficiencies in waste management practices, particularly in staff training and hazardous waste handling, the protocol emphasizes the necessity for comprehensive frameworks to ensure environmental sustainability and public health safety in healthcare settings. Through systematic risk assessment and prioritization using FMEA, critical failure modes within medical waste management are proactively identified, enabling the formulation of robust risk management strategies and mitigation measures. The protocol's step-by-step description includes preliminary investigation, department recognition, HSE risk identification, FMEA-based risk assessment, risk level determination, and final disposal methods. A case study conducted at Urmia Hospital in northwest Iran revealed specific health and safety risks associated with various waste management stages, highlighting the transfer stage as particularly critical. Inadequate training, low awareness levels, and staff shortages were identified as significant contributors to these risks. Overall, this protocol serves as a valuable resource for researchers and practitioners, facilitating the development of enhanced operational protocols and advanced safety measures in hospital waste management, ultimately improving public health outcomes and environmental protection.

## Protocol validation

Standardized data collection protocols were employed to minimize errors and ensure uniformity. Quality control (QC) measures were introduced to independently verify the accuracy of RPN calculations. Regular QC audits were conducted to ensure the proper application of the RPN formula.

By categorizing QA and QC into separate sections, the study ensures a comprehensive approach to both ensuring quality and controlling it throughout the materials and methods utilized in the evaluation of hospital waste management.

## Limitations

Subjective risk assessments may introduce bias, while resource constraints could hinder protocol implementation. Additionally, reliance on historical data may overlook emerging risks, warranting adaptations for regional regulations.

## CRediT authorship contribution statement

**Towhid Dadashi:** Writing – original draft. **Saeed Hosseinpoor:** Formal analysis. **Amir Mohammadi:** Project administration.

## Declaration of Competing Interest

The authors declare that they have no known competing financial interests or personal relationships that could have appeared to influence the work reported in this paper.

## Data Availability

The data that has been used is confidential.
